# Beneficial Features of a mHealth Asthma App for Children and Caregivers: Qualitative Study

**DOI:** 10.2196/18506

**Published:** 2020-08-24

**Authors:** Misa Iio, Yumiko Miyaji, Kiwako Yamamoto-Hanada, Masami Narita, Mayumi Nagata, Yukihiro Ohya

**Affiliations:** 1 College of Nursing Kanto Gakuin University Yokohama Japan; 2 Allergy Center National Center for Child Health and Development Setagaya Japan; 3 Division of Allergy Tokyo Metropolitan Children’s Medical Center Fuchu Japan

**Keywords:** children, caregivers, asthma, mobile app, proposed beneficial features

## Abstract

**Background:**

mHealth and uHealth apps are available for children with asthma and their caregivers. However, previous studies on mHealth apps for children older than 7 years old with asthma are limited, and most studies on asthma apps do not consider interactions involving communication between children and caregivers. Therefore, a prototype mHealth child asthma app was developed for children and their caregivers, with features of tailored feedback messages in continuing self-management and interactions between children and caregivers.

**Objective:**

The aim of this study was to identify the beneficial features of a prototype mHealth app developed for children with asthma and their caregivers.

**Methods:**

Children diagnosed with persistent asthma by allergy specialists at the National Center for Child Health and Development were recruited. The features of a prototype mHealth app for children with asthma and their caregivers were investigated using semistructured interviews after they tried the app. Data were analyzed using thematic analysis. Content-characteristic words were named and grouped together as categories to explore themes.

**Results:**

We recruited 27 children with asthma aged 2 to 12 years and 26 their caregivers. Findings on the good aspects of the app for children older than 7 years old and caregivers suggested 4 themes (confirmation of asthma knowledge, child-caregiver interaction, design of the app, and child’s interest), and 6 categories were identified. Findings on the good aspects of app for children 7 to 12 years old and caregivers suggested 5 themes (new knowledge, manga as a Japanese-style comic, child’s interest, trigger of self-management, and design and operability), and 11 categories were identified. Findings on the beneficial features of app suggested 6 themes (asthma knowledge, elements for continuous, universal design, notification, monitoring, and functions), and 12 categories were identified.

**Conclusions:**

Children with asthma and their caregivers perceived that the good aspects of the app were learning asthma knowledge with fun, including manga; interaction between child and caregiver; and easy-to-read design, such as colors. They wanted not only the asthma knowledge but also the universal design and enhanced elements, monitoring, and notification functions of the app.

## Introduction

As mobile technology and smartphones become widely used in the health care sector, several mobile medical and mobile health (mHealth) apps are being released—mHealth apps are most commonly developed to monitor a specific health disorder, to make medication management easier, or to inform users about health information and a specific health disorder. mHealth asthma apps are valuable assets for patients and caregivers alike because they offer immediate communication between patients and those responsible for providing care for their needs [1]. The asthma app’s features include asthma education material, symptom forecast, asthma action plan, telemedicine, local specialist connection, what to do in an emergency, symptom monitoring, airborne trigger identification, and clinic notifications [1]. Contents of mHealth apps for asthma since 2011 have included comprehensive information and targeting specific skills, such as the use of action plans, recommended self-care procedures, inhaler technique, and inhaler instructions [2]. The mHealth app features were classified into 7 categories (inform, instruct, record, display, guide, reminders or alert, and communicate) [3]. The most commonly used behavior change techniques of the asthma management apps were instruction, behavior health association, self-monitoring, feedback, teaching to use prompts or cues, consequences, and others’ approval [4].

A review [5] of mHealth apps for asthma in 2014 showed that 206 apps were available for patients with asthma, and 16 apps were available for children with asthma and their caregivers. There were 8 previous studies of smartphone apps to encourage asthma self-management in adolescents [6]. In pediatric asthma apps, children with asthma aged 6 to 16 years who utilized electronic adherence monitoring with daily reminder alarms together with the clinic’s feedback regarding their inhaled corticosteroid use required significantly fewer courses of oral steroids and fewer hospital admissions than those who were in the usual care arm with adherence monitoring alone [7]. One self-management app contained reminder and notification features, such as tracking of medication, for young people with asthma (aged 15 to 24 years), which resulted in high satisfaction with usefulness and ease of use [8]. Another app determined the asthma phenotype in children through the asthma control test to monitor activity, sleep, peak expiratory flow, and indoor air quality [9].

Children with chronic conditions and their caregivers need to maintain self-management behavior for symptom control, and behavior change techniques have been incorporated to support their behavior continuity. A nonuniform and individualized support was tailored as per the patient’s situation and behavior factors [10]. A meta-analysis [11] of tailoring studies that utilized print messages concluded that tailored interventions are more effective than nontailored ones. Tailoring is defined as “any combination of strategies and information intended to reach one specific person, related to the outcome of interest, and derived from an individual assessment [12].” Tailoring approaches to education have been referred to as “individual tailoring [13]” or “computer tailoring [13]” To date, tailoring has been mainly been provided through web-based programs. However, recently, tailored apps have been developed, and studies have demonstrated the effects of tailoring on the medication adherence of children with asthma [14].

A systematic review of digital asthma self-management intervention interactions since the 2000s has been reported, and interventions were mostly implemented at age 7 to 17 years [6,15]. In addition, because children who have their own smartphones are often older than 12 years, participants in most studies on pediatric asthma apps are commonly adolescents older than 12 years [16,17]. Although digital self-management programs are available for children with asthma and their caregivers, few studies have investigated the effectiveness of these mHealth apps. Moreover, most studies on asthma apps did not consider interactions involving communication between children and caregivers. Furthermore, previous studies on mHealth apps associated with asthma self-management in 7- to 12-year-old children were limited. Therefore, we developed a prototype mHealth child asthma app for children and their caregivers, including features of tailored feedback messages in continuing self-management and interactions between children and caregivers.

This study aimed to identify the beneficial features of a prototype mHealth app under development for children with asthma and their caregivers. The findings of this study are important to help perfect this app and complete a mHealth child asthma app for children and their caregivers.

## Methods

### Prototype Development

Review of mHealth apps for adolescents with chronic conditions revealed a paucity of evidence-based apps, in contrast to the thousands of apps available on the app market that are not evidence-based or user or professional informed [18]. A prototype tailored mHealth asthma app was developed based on a past tailored program using touch-screen computer [19]. The social cognitive theory [20] and tailoring [10] were used to develop a new mHealth child asthma app. This is useful in children with insufficient self-management ability because their communication levels are developmentally immature. The interpersonal approach to education had a greater effect than the tailored approach [10]; however, establishing a one-on-one relationship is difficult. Therefore, this program used the tailored approach to communication as it was most similar to the interpersonal approach. Individual interviews with caregivers were used to collect information and develop tailored messages. The prototype app was developed based on tailored program issues from past research [19] that did not involve children with asthma and their caregivers in their app design.

The mHealth child asthma app comprised a combination of asthma knowledge, behavior change, target behaviors, and theory applications. There were 3 main goals for this app: to increase caregivers’ feelings of self-efficacy in their asthma management, to increase asthma knowledge, and to continue self-management behavior of controlling asthma symptoms.

**Figure 1:**
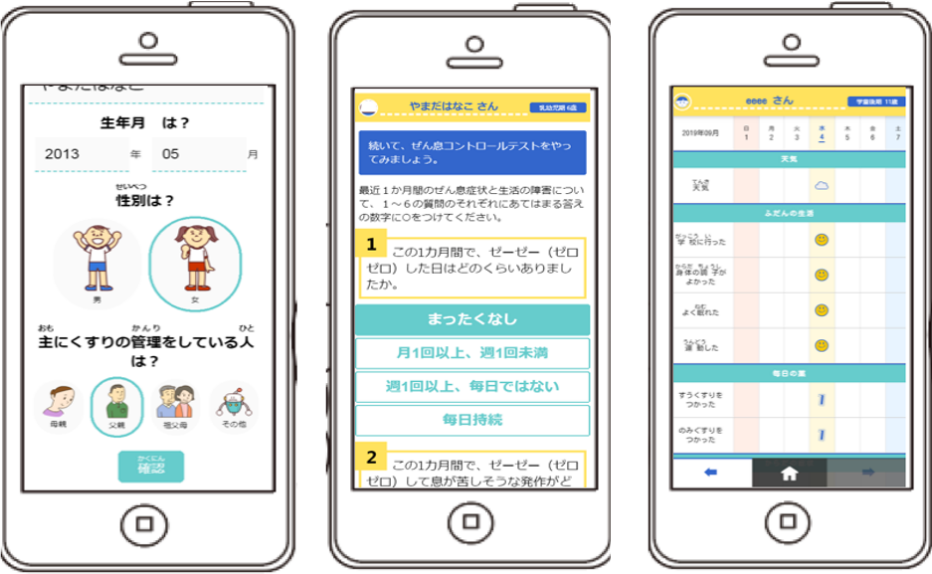
Examples of a mobile asthma app for children and their caregivers: screens commonly used for children <12 years old; setting screen (left), childhood asthma control test (middle), and self-monitoring (right).

The app’s protocols comprised 3 content areas: asthma knowledge, monitoring symptoms, and behavior change (Figure 1). This app was developed for infants and toddlers aged 0 to 6 years, school-going children aged 7 to 9 years and 10 to 12 years according to their developmental stages. Contents of the child asthma app for children aged 0 to 6 years and their caregivers were asthma knowledge (pictorial book about asthma clinical condition, causal factors, and complicating factors; quiz), self-monitoring of medications and symptoms, and tailored feedback according to the Japanese pediatric asthma control test [21]. Contents of this app for children aged 7 to 12 years and their caregivers were asthma knowledge (manga as a Japanese-style comic regarding asthma, medication, exercise-induced asthma, and stress management; quiz), self-monitoring of medication and symptoms, and tailored feedback according to the Japanese pediatric asthma control test.

### Study Design

A qualitative study was conducted, and data were analyzed using thematic analysis [22]. As part of the study, we conducted semistructured interviews, and their narratives were analyzed.

### Recruitment

Children participants aged 0 to 12 years diagnosed with persistent asthma by allergy specialists at the National Center for Child Health and Development were recruited. The types of persistent asthma, such as severity, treatment regimen, and treatment duration, were not considered. Participants were excluded if their involvement was deemed inappropriate by the pediatrician because of their mental and physical conditions. We used purposive sampling, which gathers information-rich cases that manifest the phenomenon under investigation. Participants who matched the inclusion criteria were selected by their electronic medical record. Researchers approached caregivers of children with asthma who regularly visit the hospital through telephone. A total of 31 caregivers who met the inclusion criteria by telephone were contacted. After obtaining the temporary consent to participate in the study over the telephone, caregivers and children with asthma provided consents during the next outpatient visit.

### Data Collection

We contacted pairs of children with asthma and caregivers. Participants received a mobile phone to demonstrate the prototype of the child asthma app and tried the app for 10 to 15 minutes in the interview room during their outpatient visit. After the prototype app trial, each participant was interviewed once for approximately 7 to 22 minutes (average 14 minutes). Interviews were conducted in an outpatient private room by one researcher, a nurse specializing in pediatric allergies from March 2019 to August 2019.

Data were gathered through semistructured interviews. An interview guide that matched the study aim was prepared. The researcher presented the interview guide and items to participants. The first author (MI), a researcher trained to carry out qualitative interviews, conducted all interviews. During each interview, the interviewer encouraged participants to talk freely about the proposed beneficial features of the app based on their experiences and provided opportunities to express features they felt had not been covered. The interviewer had no prior relationship with the participants.

Demographic information of participants, such as age, sex, age at onset of asthma, and relationship with caregivers, was collected. Interview contents were assessed using 4 viewpoints for each caregiver and children aged 7 to 12 years: (1) good aspects of the app, (2) proposed beneficial features of child asthma app, (3) notification (alert function) frequency and usage frequency, and (4) specific improvements of the app. Additional demographic information, such as age of asthma onset and treatment duration, was also collected. The app trial was conducted once for approximately 10 to 15 minutes before the interview; thereafter, these viewpoints points were mainly discussed to identify the portion of the app that needed modification and grasp the proposed beneficial features of the app. The interview guide was confirmed among 5 researchers before the study. The interview was recorded using a digital voice recorder after obtaining the assent of participants.

### Data Analysis

All recorded data were transcribed verbatim in Japanese. A thematic analysis was used to identify codes and themes (subcategories and categories) from the qualitative data [20]. Thematic analysis can be used to develop a novel theoretical framework and identify the common meanings and themes of an existing model. The qualitative data were analyzed in 4 phases: (1) becoming familiar with the collected data, (2) generating the codes and collating similar data for each code, (3) naming content-characteristic words and grouping them as categories, and (4) exploring themes agreed upon by 4 research members who engaged in consensus decision making. Each theme in this study was derived from interview data. Research members included 2 pediatric nurses (MI and MN) and 3 pediatricians specializing in allergies (YM, KY, and MN). After initial coding of each transcript, the researchers discussed and identified a set of main themes, categories, and subcategories.

### Informed Consent/Assent and Ethical Considerations

The Japanese National Center for Child Health and Development committee for ethics in research of social medicine (approval number: 2028) and the university committee for ethics in research involving human subjects approved this study. Participants who met the inclusion criteria together with their caregivers were informed verbally and in writing about the aim, significance, and methods of the study. They were informed of their rights as voluntary participants, including the right to withdraw from the study, data anonymity, protection of confidential information, handling and disposal of data, and the possibility of results being published. After receiving this information, participants 7 to 12 years old provided informed written assent, and caregivers of all participants provided written consent. COREQ guidelines [23] were followed.

## Results

### Participant Demographics

Out of the total 31 caregivers who were contacted by telephone, 4 caregivers of 1- to 12-year-old children (2 girls and 2 boys) did not participate because they were unable to contact us by telephone several times, and it was difficult to secure the time of the outpatient visit. A total of 27 pairs of caregivers and children were interviewed. Participant characteristics are shown in Table 1. Participants were divided into the developmental stage groups: children younger than 7 years old (n=10) and children 7 to 12 years old (n=17). The majority of caregivers were between the ages of 40 and 49 years. Two viewpoints in the prototype of the child asthma app were identified: good aspects of the app and proposed beneficial features for the app.

**Table 1:** Participants’ characteristics.

Participant characteristics				Participants, n (%)		
**Patient**				**27 (100)**		
	**Developmental stage (years)**					
		**Preschool children**				
			2-3			3 (11)
			4-6			7 (26)
		**School-aged children**				
			7-9			9 (33)
			10-12			8 (30)
	**Sex**					
		Boy			18 (67)	
		Girl			9 (33)	
	**Age of onset (years)**					
		0-2			14 (52)	
		3-5			9 (33)	
		6-8			4 (15)	
**Caregiver^a^**				**26 (100)**		
	**Relationship**					
		Mother			25 (96)	
		Father			1 (4)	
	**Age (years)**					
		30-39			8 (31)	
		40-49			17 (65)	
		50-59			1 (4)	

^a^Patients included 2 sets of siblings.

### Good Aspects for Caregivers of Children younger than 7 Years Old With Asthma

#### General

Coding and classifying phases revealed 6 categories and 4 themes from 25 codes in *good aspects of the app for the caregivers of children younger than 7 years old with asthma* (Table 2). By grouping the categories, 4 themes (ie, confirmation of asthma knowledge, child–caregiver interaction, app design, and child’s interest) were identified.

**Table 2:** Good aspects for children younger than 7 years old with asthma and their caregivers.

Themes and categories		Representative caregivers’ verbatim comment
**Confirmation of asthma knowledge**		
	Reconfirmation of asthma knowledge	“I was able to re-recognize my knowledge with the quiz.” [40s, mother of a 5-year-old girl]
	Acquisition of asthma knowledge	“I learned quiz knowledge.” [40s, mother of a 2-year-old girl]
		“I heard from my pediatrician that I realized that I should be careful about daily life and playing outside.” [40s, mother of a 5-year-old girl]
		“I don't seem to have knowledge, or I know it, but there are things I don't know, so if it's a quiz format, I think it's easy to understand and increase knowledge.” [40s, mother of a 5-year-old boy]
**Child–caregiver interaction**		
	Initiatives for parents and children	“Since the child still does not know about asthma, the app was good for the parent and child to do it together.” [30s, mother of a 4-year-old girl]
		“Although it is a difficult content such as the asthma mechanism, there is an illustration and the child became aware of asthma.” [30s, mother of a 4-year-old girl]
**Design**		
	Ease of viewing the screen	“I thought app was easy to understand because of illustrations.” [40s, mother of a 5-year-old boy]
		“The size of quizzes was good.” [30s, mother of a 6-year-old boy]
	Presence of childhood asthma app	“The good thing was that there was an asthma app.” [40s, mother of a 2-year-old boy]
**Child’s interest**		
	Child’s interest	“The child just got interested in the body and bought a picture book, so I thought the app would be fun in order to ask questions about the structure that rubs the coughing part.” [40s, mother of a 4-year-old girl]

#### Theme 1: Confirmation of Asthma Knowledge

The coding and classifying phases revealed 2 categories in this theme. Caregivers of children younger than 7 years old with asthma reported good aspects including *reconfirmation of asthma knowled*ge and *acquisition of asthma knowledge*. They reported that they learned and recognized asthma knowledge through the app.

#### Theme 2: Child–Caregiver Interaction

**Figure 2:**
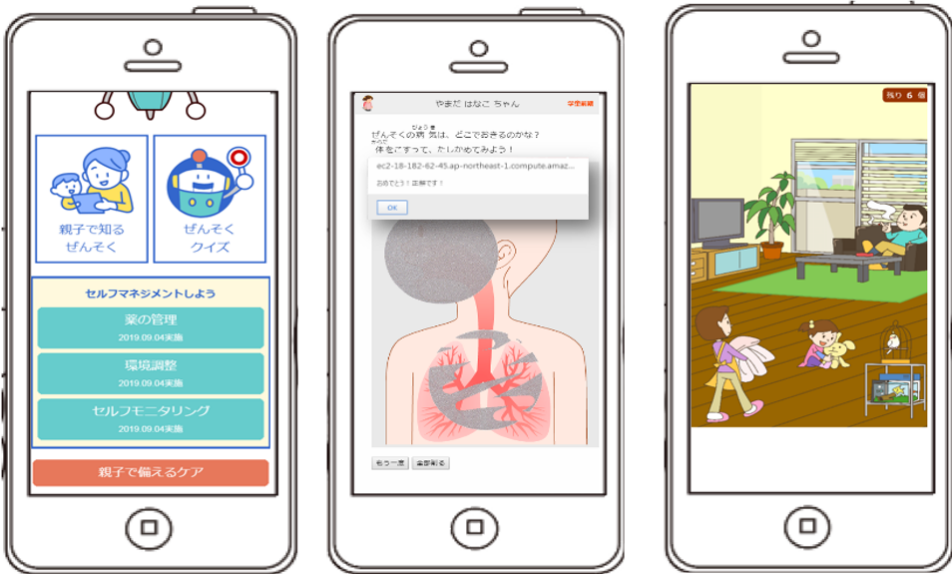
Examples of features for children <7 years old with asthma and caregivers: main home screen (left) and asthma pictorial book with scratch and search for asthma deterioration factors (middle and right).

The coding and classifying phases revealed 1 category in this theme. Caregivers of children younger than 7 years old with asthma reported good aspects including *initiatives for parents and children*. They reported that the picture book helped ascertain asthma knowledge with the child younger than 7 years old, creating a positive child–caregiver interaction (Figure 2).

#### Theme 3: Design

The coding and classifying phases revealed 2 categories in this theme. Caregivers of children younger than 7 years old with asthma reported good aspects including *ease of viewing the screen* and *presence of childhood asthma app*. They reported that viewing the screen was easy and the font size was good. In addition, as the number of mobile child asthma apps is very limited in Japan, having this newly developed app was a good way to manage children’s asthma.

#### Theme 4: Child’s Interest

The coding and classifying phases revealed 1 category in this theme. Caregivers of children younger than 7 years old with asthma reported good aspects including *child’s interest*. They reported that it was good that children 7 years old could learn with the caregiver as they began to be interested in how the body functions.

### Good Aspects for 7- to 12-Year-Old Children With Asthma and Their Caregivers

#### General

The coding and classifying phases revealed 11 categories from 50 codes for *good aspects of the app for 7- to 12-year-old children with asthma and their caregivers* (Table 3). By grouping the categories, 5 themes were identified: new knowledge, manga, child’s interest, app design, self-management promotion, and app operability.

**Table 3:** Good aspects for 7- to 12-year-old children and caregivers.

Themes and categories		Representative verbatim comment
**New knowledge**		
	Acquisition of asthma knowledge	“The quiz gave me the knowledge I expected.” [child aged 7, boy]
	Specific explanation	“The app was easy to understand that I used to explain each difficult word.” [child aged 12, boy]
	New discovery	“Even though I knew the app, there was a new discovery (I thought I would die only through suffocation, but I knew that I usually suffer from a wound in the bronchi).” [child aged 9, boy]
**Manga**		
	Manga	“I'm tired of writing alone, but I thought that manga was easy to understand.” [child aged 12, girl]
		“Because the app for asthma knowledge was a manga, my child read it with interest.” [40s, mother of a 7-year-old boy]
		“Manga was interesting.” [child aged 10, boy]
		“It was easy to understand because there was manga that understood asthma well.” [child aged 9, boy]
		Colored manga was easy for children to be interested in, and my child was also interested. [40s, mother of a 10-year-old boy]
**Child’s interest**		
	Child’s interest	“My child was having fun.” [40s, mother of a 7-year-old boy]
		“I thought I could play with the app.” [child aged 9, boy]
		““My child likes playing with mobile phones, so I thought it would be interesting to do something with mobile phones.” [40s, mother of a 7-year-old boy]
**Trigger of self-management**		
	Opportunities for child self-management	The child still does not want to manage it with a paper diary; therefore, the app is an opportunity to do it himself. [40s, mother of a 7-year-old boy]
	Opportunities for parent-child communication	“While checking with karuta [Japanese card game], I thought that the app would allow a proper communication between parents and children.” [30s, mother of a 9-year-old boy]
**Design and operability**		
	The color of the illustration	“The color is good and easy-to-read.” [child aged 10, boy]
		“I thought the color of manga was really beautiful.” [40s, mother of a 7-year-old boy]
	Character size	“The size of the characters and kanji [Japanese-characters] was fine.” [child aged 9, boy]
		“I could read the size of the character at karuta.” [40s, mother of a 12-year-old girl]
		“The size of the letters could be large even if small.” [child aged 10, boy]
	Ordinariness	“The app was ordinariness.” [child aged 7, boy]
	Easy input operation for monitoring	“Asthma diary had to be issued individually, but the app was easy to use.” [30s, mother of an 8-year-old boy]
		“The monitoring operation was easy.” [30s, mother of an 8-year-old boy]

#### Theme 1: New Knowledge

The coding and classifying phases revealed 3 categories in this theme. Children aged 7 to12 years with asthma and their caregivers reported good aspects including *acquisition of asthma knowledge*, *specific explanation*, and *new discovery*. They reported that the app had provided asthma knowledge and they gained new information on childhood asthma.

#### Theme 2: Manga (Japanese-Style Comic)

**Figure 3:**
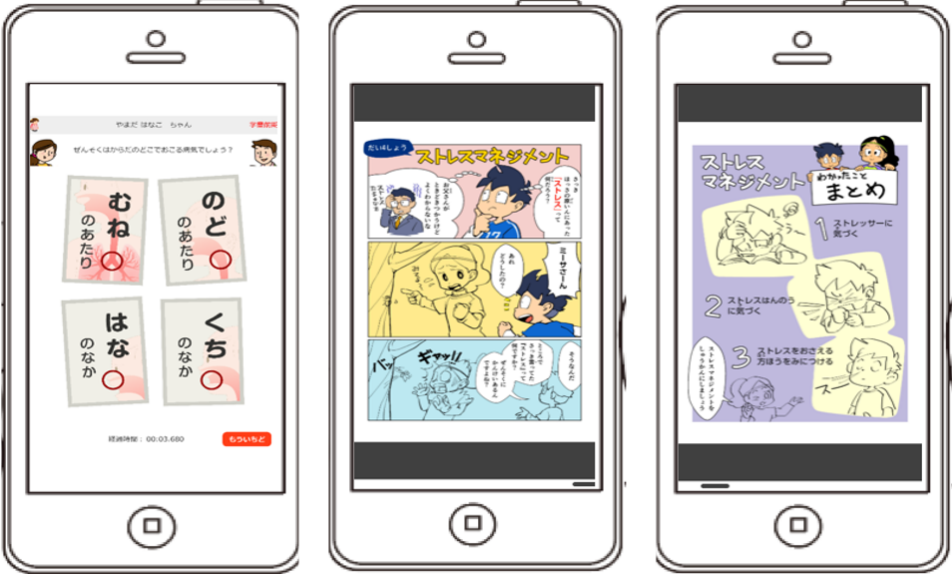
Examples of the features of asthma knowledge for 7- to 12-year-old children: asthma quiz (left) and manga about stress management of asthma (middle and right).

The coding and classifying phases revealed 1 category in this theme. Children aged 7 to 12 years with asthma and caregivers reported good aspects including *manga about child asthma*. They reported that they enjoyed learning about asthma using manga (Figure 3).

#### Theme 3: Child’s Interest

The coding and classifying phases revealed 1 category in this theme. Children aged 7 to 12 years with asthma and their caregivers reported good aspects including *child’s interest*. Caregivers reported that children had fun with the app, and 7- to 12-year-old children with asthma stated that they could play with the app.

#### Theme 4: Self-Management Promotion

The coding and classifying phases revealed 2 categories in this theme. Children aged 7 to 12 years with asthma and their caregivers reported good aspects including *opportunities for child self-management* and *opportunities for parent–child communication*. Caregivers reported that the app provided opportunities for the child’s self-management and child–caregiver communication.

#### Theme 5: Design and Operability

The coding and classifying phases revealed 4 categories in this theme. Children aged 7 to 12 years with asthma and caregivers reported good aspects including *color of the illustration*, *character size*, *ordinariness*, and *easy input operation for monitoring*. They reported that the color of the illustration about manga and character size were good. Children aged 7 to 12 years with asthma stated that the app was not ordinary. In addition, caregivers reported that the operation of asthma diary and monitoring were easy.

### Proposed App Features From the Perspective of Child or Caregiver

#### General

The coding and classifying phases revealed 44 subcategories and 12 categories from 214 codes in *proposed beneficial features for the app for children with asthma and their caregivers* (Table 4). By grouping the categories, we explored 6 themes: asthma knowledge, elements for continuity, universal design, monitoring, and functions.

**Table 4:** Proposed beneficial features for the app in children with asthma and caregivers.

Themes and categories		Subcategory	Developmental stage		
			2-6 years	7-9 years	10-12 years
**Asthma knowledge**					
	Asthma knowledge	Asthma knowledge in general	✓	✓	✓
		Specific behavior of management		✓	
	Regular provision of asthma knowledge	Regular provision of knowledge		✓	
		Newsletter		✓	
**Elements for continuous**					
	Ideas that children can learn	Quiz	✓	✓	✓
		Manga		✓	✓
		Devices that can be used by younger children	✓		
		Sound/video	✓		✓
		Amusement	✓	✓	
		Character	✓		
	Devices that can be used continuously	Game element		✓	✓
		Incentive for continuing		✓	
		Clear the stage/rank up			✓
		Increase self-efficacy	✓	✓	
		Update the information	✓		
		Not game element		✓	
		Consideration of developmental stage			✓
		Devices that do not end with a single use	✓		
		Clarification of what is ahead	✓	✓	
		Devices that can be used in the next step			✓
	Character input for 7-12 years old children	Possible		✓	✓
		Impossible by developmental stage		✓	✓
**Universal design**					
	Universal design	Illustration/color shade		✓	✓
		Visibility/readability			✓
**Notification**					
	Notification function	Necessity of reminder notification	✓		✓
		Message notification function	✓		
	Notification frequency	Every day at a fixed time	✓	✓	✓
		No notification required	✓	✓	
		Self-configuration	✓	✓	
		Notification once a week		✓	
**Monitoring**					
	Monitoring function	Function of symptom input	✓	✓	✓
		Easy operation			✓
		Calendar function		✓	
		Remaining drug notification		✓	
		Remarks column settings		✓	
		Unnecessary	✓	✓	
		Self-configurable monitoring items		✓	
	Monitoring frequency	Daily input	✓	✓	✓
		Entering all at once (eg, entering once a week)	✓		
**Functions**					
	Used by 7-12 years old children alone	Use of apps by children alone		✓	✓
	Operability and functions	Functions of the app	✓	✓	✓
		Family (sibling or parent) function	✓		
		Response	✓		
		What does not take time	✓		

#### Theme 1: Asthma Knowledge

The coding and classifying phases revealed 2 categories in this theme. Children with asthma and caregivers reported proposed beneficial features for the app including *asthma knowledge* and *regular provision of asthma knowledge*. They had needs for asthma knowledge in general knowledge and specific behavior management and the regular provision of asthma knowledge.

#### Theme 2: Elements for Continuity

The coding and classifying phases revealed 3 categories in this theme. Children with asthma and caregivers reported proposed beneficial features of the app including ideas that children can learn, *devices that can be used continuously* and character input for 7- to 12-year-old children. They expressed the need for fun elements for continuous use of the app, such as quiz, manga, game element, clear the stage, and so on.

#### Theme 3: Universal Design

The coding and classifying phases revealed 1 category in this theme. Children with asthma and caregivers reported proposed beneficial features of the app including *universal design*. They expressed the need for universal design, such as illustration or color shade and visibility or readability, that were easy to use for everyone, from children to adults.

#### Theme 4: Notification

The coding and classifying phases revealed 2 categories in this theme. Children with asthma and caregivers reported proposed beneficial features of the app including *notification function* and *notification frequency*. They expressed the need for message notification function and necessity of reminder notification every day at the fixed time or once a week or no notification.

#### Theme 5: Monitoring

The coding and classifying phases revealed 2 categories in this theme. Children with asthma and their caregivers reported proposed beneficial features of the app including *monitoring function* and *monitoring frequency*. They expressed the need for the easy operation, remaining drug notification, and daily input. In addition, a mother (in her 40s) of a 7-year-old boy reported that monitoring with the app can be shared with the pediatrician during their outpatient visit.

#### Theme 6: Functions

The coding and classifying phases revealed 2 categories in this theme. Children with asthma and caregivers reported proposed beneficial features of the app including *use by 7- to 12- year-old children alone* and *operability and functions*. Children aged 7 to 12 years generally do not own a mobile phone; therefore, they need to be able to use one. In addition, children with asthma and their caregivers needed operability and functions, such as family (sibling or parent) function among others.

## Discussion

### Principal Results

Four themes (confirmation of asthma knowledge, child-caregiver interaction, app design, and child’s interest) comprising the good aspects of the app were identified for children older than 7 years old with asthma and their caregivers. Moreover, 5 themes (new knowledge, manga, child’s interest, app design, self-management trigger, and app operability) comprising the good aspects of the app for 7 to 12 years old children with asthma and their caregivers were also determined. In addition, 6 themes (asthma knowledge, elements for continuous, universal design, monitoring, and functions) on the proposed beneficial features of the app were identified for children with asthma and their caregivers.

### Proposed Beneficial Features for an mHealth Child Asthma App

Children with asthma and their caregivers captured the features of asthma knowledge as good aspects of the app. On the one hand, they wanted elements of continuity in the app, such as quiz, manga, and games, among others. Hospital outpatients identified requiring more knowledge about the diseases through the smartphone app [24]. In addition to providing asthma knowledge, ways should be devised for 7- to 12-year-old children with asthma and even their caregivers to learn asthma knowledge while continuously accessing the app. Manga about asthma in this study was highly acclaimed by 7- to 12-year-old children with asthma. The concept of entertainment education [25], entertainment media that provide educational information intended to increase psychological readiness toward the desirable behavior change, such as television, digital games, and comics, has potentially become an effective communication strategy to implement the health message for the high-risk population [26]. Several educational programs have adapted manga as an effective learning tool, known as an educational manga [27,28]. Additionally, previous studies [29,30] challenged the application of manga or comic book for patient education targeting children. By incorporating Japanese culture manga into the app, 7- to 12- year-old children can easily accept new information that results in high educational effects. Serious games without entertainment, enjoyment, or fun as their primary purpose [31], have emerged as a new generation of videogames meant to provide education and training [32]. Although serious games designed for asthma education have evolved with advances in technology, results of their evaluation remained similar across studies, with clear improvements in knowledge but little to no change in behaviors and clinical outcomes [33]. Therefore, although game elements were useful in terms of gaining asthma knowledge, it was suggested that they were unsuitable for self-management and behavior change.

Two themes that were good aspects of this app were the child’s interest and design and operability, which were commonly reported by 7- to 12-year-old children with asthma and their caregivers. Additionally, findings on the proposed beneficial features of the app suggested that children with asthma and their caregivers wanted a universal design that could easily be used by everyone. The design and development of a self-management app included validation of app features through user-centered design methods [34,35]. The patient-centered universal design of the app should also be improved, and its feasibility determined. Furthermore, caregivers of children with asthma should also enhance the basic functions and operability, not complex functions. Children aged 7 to 12 years with asthma and their caregivers required notifications, reminders, monitoring, and alerts. This supports the findings of previous studies [36] on asthma management mHealth app for adolescents. In addition, caregivers' needs for app monitoring were to be able to share the monitoring content with their pediatrician during their outpatient visits. The sharing of symptom monitoring in children's daily lives promotes communication between children with asthma and their caregivers and pediatricians and maintains good relationships. Moreover, sharing of monitoring content helps pediatricians make treatment decisions by understanding the appearance of detailed asthma symptoms of the children. The monitoring and alerting functions should be developed not only for the ease of use but also for the pace of the user. In future app improvements, ways to set monitoring items and notifications by the users themselves should be devised. In particular, 7- to 12-year-old children with asthma wanted to keep records in monitoring while having fun. As for the monitoring function of children with asthma, incentives obtained by the child’s continuous medication intake and input symptoms are necessary.

### Limitations

This study had several limitations. Findings derived from 27 children and 26 caregivers at one children’s hospital were used to identify the proposed beneficial features of the app in children with asthma and their caregivers and are limited to that population. In addition, the majority of participants were boys and the majority of caregivers were mothers. Pediatric asthma has a high prevalence in boys in Japan, as is true worldwide. Furthermore, although the number of double-income families is increasing in Japan, mothers still attend the outpatient visit of children. However, in Japan, patients who go to children’s hospital are those with severe asthma and are likely to use the app frequently for long term. Therefore, the results were useful in understanding the needs of the target population: children younger than 7 years old and 7 to 12 years old with asthma and their caregivers. In addition, this study has clarified caregiver-reported proposed beneficial features of the app that in children younger than 7 years old were not as they reported. Children less than 7 years old were having difficulty for answering the questions accurately given their limited language function and cognitive development; therefore, caregivers were asked to answer their needs.

Additionally, since school-aged children aged 7 to 12 years often do not have their own mobile phones, their caregivers’ mobile phones were used. However, as shown in the results of this study, despite the child’s inconvenience in using the app, using a caregiver’s mobile phone can lead to communication and interaction between them and their caregivers. Furthermore, the study did not determine whether the app changed the participant’s behavior. Moreover, there are very few apps for child asthma management in Japan; therefore, we did not include questions in the interview on the topics of “why they would not use an app” and “what they did not like.” We also did not consider the 4 patients who declined to participate as being any different from the participants. However, considering that the app provides potentially more information is very important. Finally, it would be worth reiterating that the app was only tried for approximately 15 minutes at the time of the interview; the conditions would be very different if they were used at home. The next step should be to develop and complete the contents and features of the app including the results of this study. To continue the self-management behavior of asthma in children with asthma and their caregivers, communication and interactions between the child and caregiver should be enhanced through the app. In addition, it is necessary to consider behavior changes associated with the app usage.

### Strengths and Future Research

One of the strengths of this study was that it included preschool children aged 0 to 6 years and school going children aged 7 to 12 years who were not previously targeted by app developers. Additionally, another strength was that it incorporated manga; therefore, children with asthma and their caregivers could have fun while learning about asthma. The app included monitoring symptoms and medication, asthma control, and tailored messages so that it could be used continuously rather than in a single sitting.

Based on the results of this study, the app was improved and completed. The completed app is now undergoing formal feasibility and usability testing in children with asthma and their caregivers. Data collection from 2 to 12 years old children, such as usage data of app features and their collection of behavior changes, will help evaluate its feasibility, usability, and operability. The process and content evaluation of the app will help identify the contents for effective patient education in preschool and school going children aged 0 to 12 years with asthma and their caregivers.

### Conclusions

Children with asthma and their caregivers perceived the good aspects of the app about learning the asthma knowledge with fun, including manga, child–caregiver interaction, contents that interest children, and easy-to-read design such as colors. Children with asthma and their caregivers wanted not only the asthma knowledge but also the enhanced monitoring and notification functions of the app, elements that could be continuously used by children, and universal design that can easily be used for everyone.
